# Anchor-Related Granulomatous Reaction After Single-Incision Mid-urethral Sling Placement: A Case Report

**DOI:** 10.7759/cureus.107413

**Published:** 2026-04-20

**Authors:** Olga Triantafyllidou, Emmanouela Angouridaki, Konstantinos Karkalemis, Fotios Vlahos, Nikolaos Vlahos

**Affiliations:** 1 Second Department of Obstetrics and Gynaecology, Aretaieio University Hospital, Medical School, National and Kapodistrian University of Athens, Athens, GRC; 2 School of Medicine, National and Kapodistrian University of Athens, Athens, GRC; 3 School of Medicine, University of Milano Bicocca, Milan, ITA

**Keywords:** foreign-body granuloma, groin pain, single-incision mini-sling, stress urinary incontinence, surgical case report, vaginal erosion

## Abstract

Urinary incontinence is a common condition in adult women, significantly affecting quality of life, daily activities, and psychosocial well-being. Stress urinary incontinence (SUI) is the most frequent subtype, and it often coexists with pelvic organ prolapse (POP), as both conditions result from pelvic floor dysfunction and share risk factors such as childbirth and menopause. The Altis® mid-urethral sling (Coloplast Corp., Minneapolis, MN, USA) is a minimally invasive mesh implant designed to treat stress urinary incontinence in women by providing urethral support. Although it demonstrates high success rates, potential complications, such as early-onset groin pain, urinary tract infection, voiding difficulties, subepithelial erosion or mesh extrusion, labial nodule, or paraurethral granuloma, may arise, sometimes requiring medical or surgical intervention. Our patient, a 43-year-old woman (P2002), developed severe early-onset groin pain, subepithelial vaginal erosion, a left paraurethral granuloma, and a labial subcutaneous nodule following the placement of an Altis® single incision sling system for stress urinary incontinence confirmed by urodynamic studies. Her symptoms began within days of the procedure. On examination, there was ulceration of the left vaginal wall with granulation tissue, though no mesh fibers were visible. MRI showed inflammation along the left sling’s anchoring arm. The patient underwent surgical excision of the granuloma, removal of the left sling arm, and resection of the labial nodule, resulting in complete resolution of her symptoms. This case highlights an uncommon early complication of single-incision mid-urethral sling placement related to anchor-associated granulomatous inflammation. Early-onset groin pain and subepithelial vaginal erosion may occur without visible mesh exposure. Prompt recognition and targeted surgical management can result in symptom resolution and favorable clinical outcomes.

## Introduction

Stress urinary incontinence (SUI) represents the most common subtype of urinary incontinence and has a significant impact on quality of life, daily functioning, and psychosocial well-being [1]. The prevalence showed substantial variation across studies, ranging from 7.9% in the study by Elks W et al. [2] to 88.6% in the study by Ferreira et al. [3]. A recent meta-analysis of the 10 included studies produced an overall pooled prevalence of 35.8%. The main mechanism of urinary incontinence during stress is an inadequate anatomical support of the bladder neck and proximal urethra in an elevated retropubic position. These structures are stabilized by a supportive tissue “hammock” extending from the anterior vaginal wall and attaching bilaterally to the pelvic diaphragm [4]. SUI frequently coexists with pelvic organ prolapse (POP), as both arise from pelvic floor dysfunction, with up to 60% of women with POP also experiencing urinary incontinence and nearly 40% of incontinent women showing POP, often due to shared risk factors like childbirth and menopause [5].

The management of stress urinary incontinence initially involves conservative measures, including lifestyle modifications, weight loss, pelvic floor muscle training, bladder training, and behavioral therapies. The use of a synthetic mid-urethral sling has been established as a standard surgical procedure for SUI in cases of failure of conservative treatment, providing effective urethral support with minimal invasiveness [6]. Tension-free transobturator tape (ΤΟΤ) was introduced by Delorme in 1991 to reduce the risk of bladder injury [7]. This sling is placed through the obturator membrane in the middle third of the urethra, providing encouraging results and cure rates of 93% [8]. Single-incision mini-slings (SIS) were developed to further minimize the morbidity associated with continence surgery. Among single-incision slings, the Altis® single incision mini-sling system (Coloplast Corp., Minneapolis, MN, USA) and conventional TOT Abbrevo® single incision sling system (Coloplast Corp.) are commonly used in our institution via a single vaginal incision, offering a transobturator approach and adjustable tension [9] However, mesh‑related complications, such as subepithelial vaginal erosion or mesh extrusion and vaginal mesh exposure or paraurethral granulomas with associated groin or pelvic pain, have been reported [10]. These complications may present with pain, palpable masses, vaginal discharge, or urinary symptoms and may require surgical revision or sling removal.

We report a case of early‑onset groin pain, subepithelial erosion, paraurethral granuloma, and labial nodule occurring shortly after Altis® sling placement, successfully managed with excision of the affected sling arm and the granulomatous tissue.

## Case presentation

**Figure 1:**
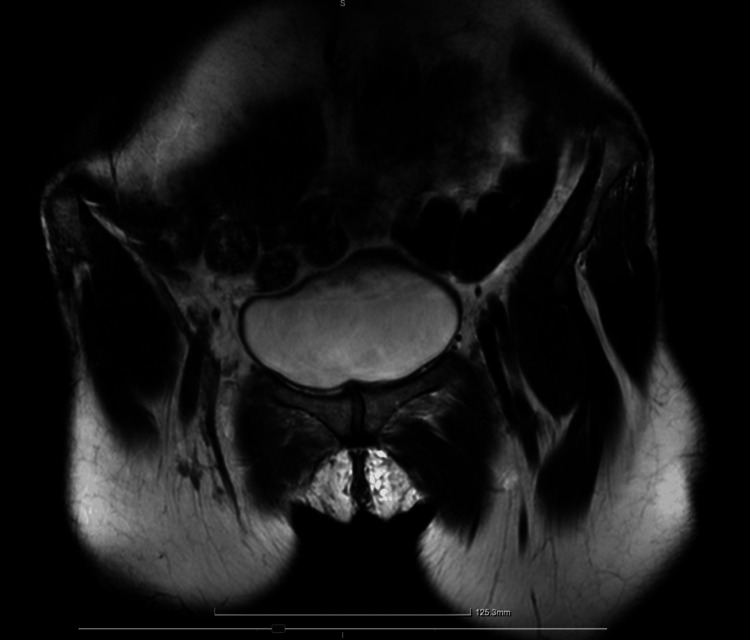
Magnetic resonance imaging (MRI) on postoperative day 4 demonstrating no evidence of periurethral injury or hematoma T2 sequence MRI without contrast enhancement

A 43-year-old woman, gravida 2, para 2 (P2002), with two prior term vaginal deliveries, presented with severe stress urinary incontinence significantly affecting her daily activities. She had no relevant comorbidities and no history of prior pelvic surgeries. Preoperative urodynamic studies confirmed genuine stress urinary incontinence, normal bladder compliance, no detrusor overactivity, and no vaginal relaxation. The patient underwent Altis® single-incision sling placement according to company specifications. The patient was discharged from the hospital the following day; however, four days later, she reported left groin pain radiating to the inner thigh, which was aggravated by walking. An MRI was performed at that time, and the radiologist confirmed that no hematoma, injury, or signs of inflammation were present and that the sling was in place (Figure 1). The patient was treated with nonsteroidal anti-inflammatory drugs (NSAIDs) with significant improvement of her symptoms. At the six-week follow-up, she reported no stress incontinence, the vaginal incision was well-healed, and the pain was improving. The patient was instructed to initiate intercourse and to report on the progress of her symptoms.

**Figure 2:**
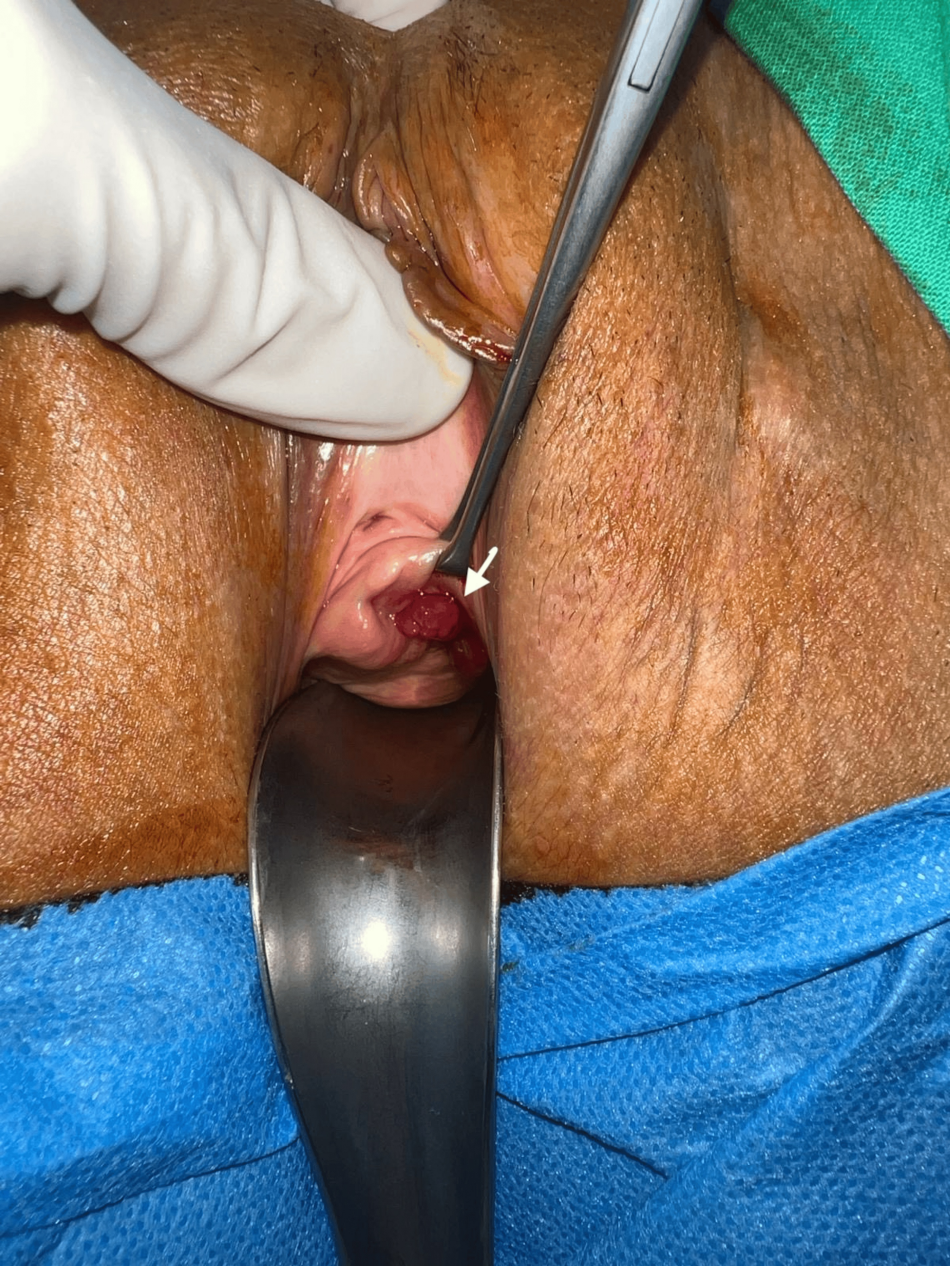
Ulceration/erosion of the left vaginal (1.5 cm) wall covered with dense granulation tissue (arrow)

**Figure 3:**
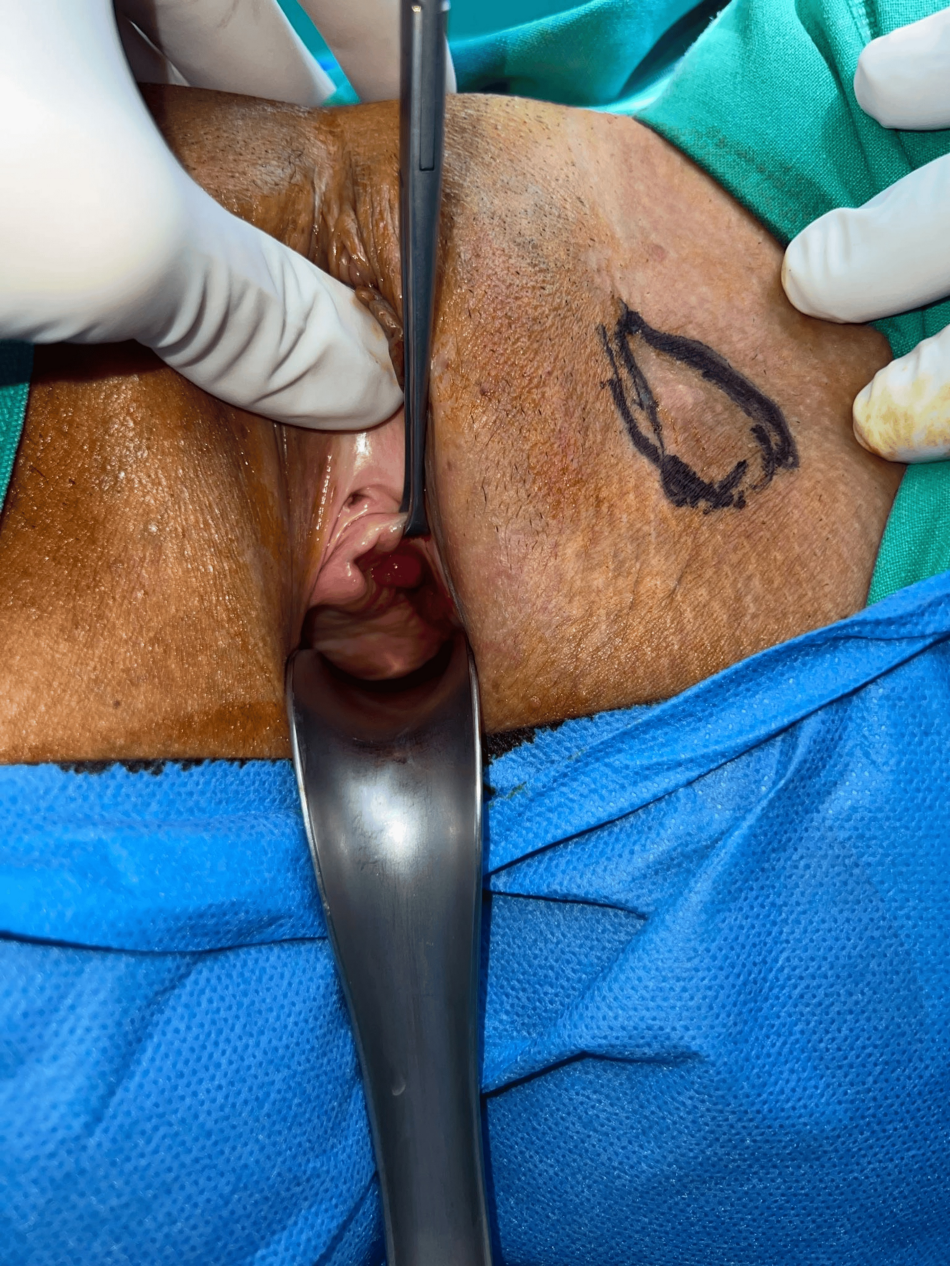
Tender subcutaneous nodule measuring approximately 3 cm in diameter, located lateral to the left labia majora The palpable lesion was delineated on the overlying skin with a surgical marker to facilitate precise intraoperative identification (circled area).

Three months later, the patient reported occasional vaginal discomfort during intercourse accompanied by spotting, a sensation of a foreign body, and subsequently significant dyspareunia. Physical examination revealed a 1.5-cm ulceration/erosion of the left vaginal wall covered with dense granulation tissue, as well as a tender subcutaneous nodule measuring approximately 3 cm in diameter, located lateral to the left labia majora (Figures 2, 3). No mesh fibers were visible. Marked tenderness over the left obturator region was noted, but there were no signs of infection or purulence. MRI demonstrated inflammation surrounding the left anchoring arm of the sling, extending from the paraurethral region to the subcutaneous tissues lateral to the left labia majora, without abscess formation. Laboratory investigations, including urine culture and systemic inflammatory markers, were normal.

**Figure 4:**
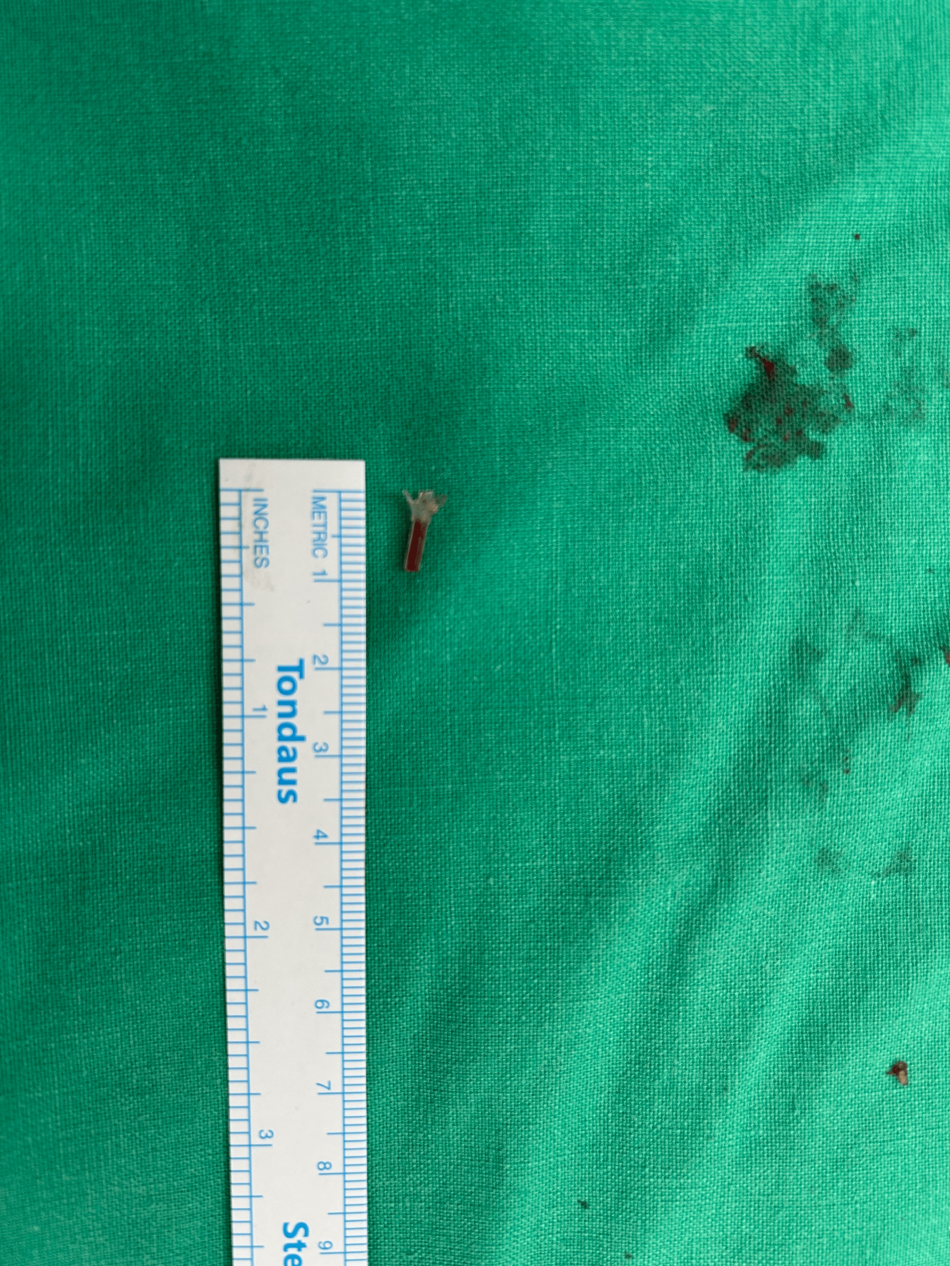
Plastic end of the Altis® sling after complete removal of the left anchoring arm

**Figure 5:**
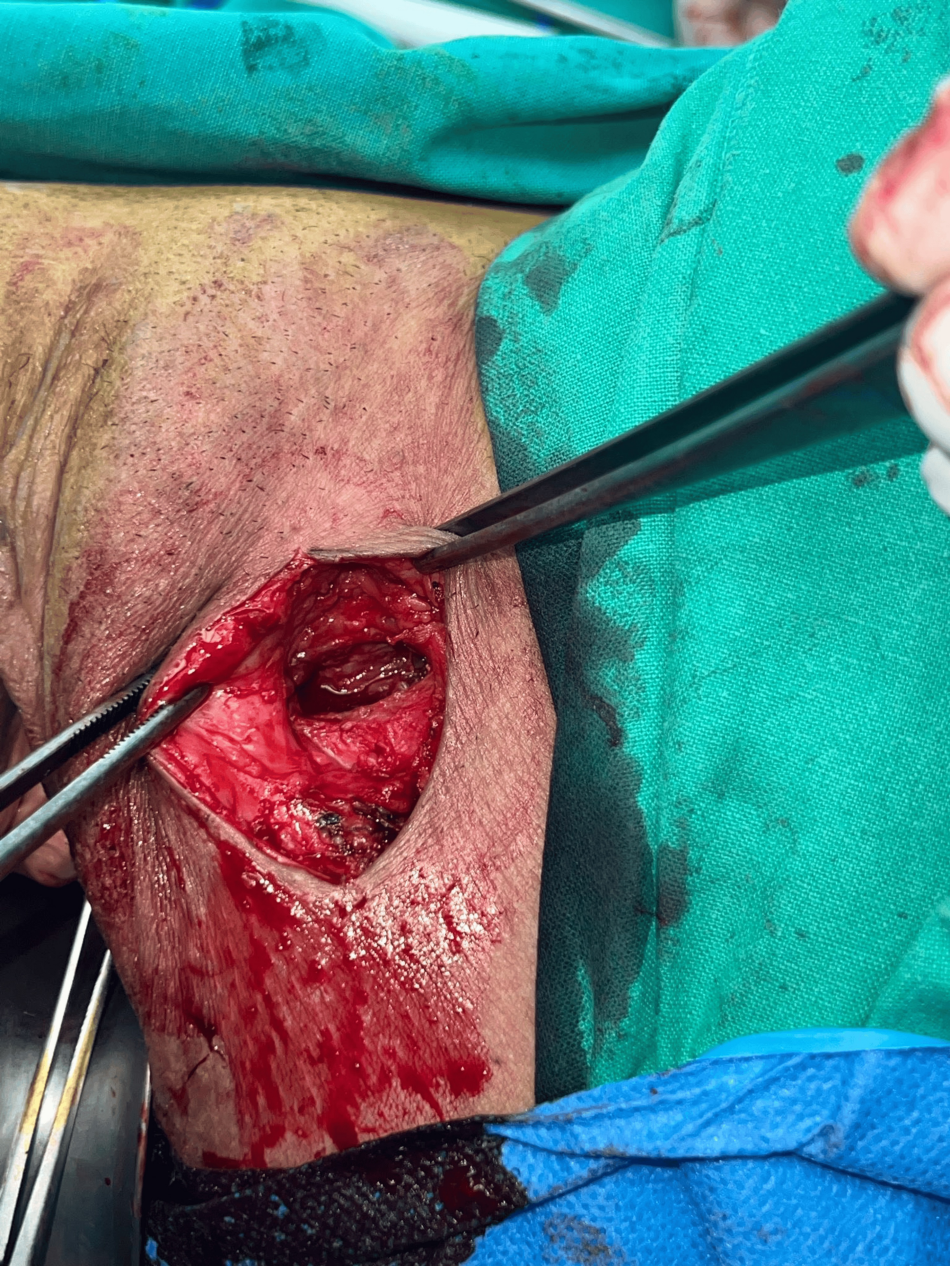
Excision of the left labial subcutaneous nodule, which was extending to the fascia of the left external obturator muscle

The patient underwent transvaginal excision of the ulcerated area and paraurethral granuloma, complete removal of the left anchoring arm of the Altis® sling with its plastic ending (Figure 4), and excision of the left labial subcutaneous nodule, which extended to the fascia of the left external obturator muscle (Figure 5). The vaginal wall was approximated with 2.0 vicryl in a single layer. The defect of the obturator fascia was repaired with interrupted #1 Vicryl, and the incision in the inner thigh lateral to the left labium majus, with a two-layer closure of the subcutaneous tissue and the skin. No operative complications occurred.

Histological microscopic examination later demonstrated foreign-body-type granulomatous inflammation characterized by multinucleated giant cells and lipophagic macrophages, features related to prior mid-urethral sling placement. No signs of infection were apparent in microscopic examination.

At the six-week follow-up, the vaginal incision and the skin incisions had healed well, and the groin pain had improved by approximately 80%. By three months, she reported complete resolution of her symptoms but only occasional mild urine leakage. Based on the 24-hour pad test, the degree of incontinence was classified as mild (2-10g/day). Urodynamic re-evaluation demonstrated mild recurrent stress urinary incontinence, which was not bothersome to the patient. The patient expressed significant improvement in quality of life.

## Discussion

Only a few cases of chronic foreign body reaction manifesting as granuloma formation after mid-urethral sling placement have been reported. For example, a groin mass developing years after a transobturator sling was identified as a granuloma surrounding the mesh [11]. Nevertheless, to the best of our knowledge, there are no published reports combining all of the following features: very early onset postoperative groin pain, subepithelial vaginal wall erosion masked by granulation tissue (i.e., without visible mesh extrusion or exposure), and a labial subcutaneous inflammatory nodule along the anchor tract.

Mesh-related complications following mid-urethral slings are well-documented. The overall cumulative incidence of reoperation following surgical treatment for urinary incontinence according to the Danish National Patient Registry is 10% [12]. The lowest reoperation rates were observed in women who underwent pubovaginal sling procedures (6%), retropubic midurethral tape placement (6%), or Burch colposuspension (6%), followed by TOT procedures (9%). Moreover, TOT procedures showed a 2-fold higher risk of reoperation (HR, 12.1; 95% CI, 1.5-2.9) compared to retropubic midurethral tape [12]. Vaginal or urethral erosion/extrusion, chronic foreign body reaction, groin or pelvic pain, and voiding dysfunction have been described, most commonly occurring months to years after surgery [11,13]. Vaginal extrusion of synthetic midurethral slings has been reported in approximately 2-3% of women [4]. Dwyer et al., in their recent review, mentioned 2.1% (11 out of 513 patients) of type extrusion after conventional TOT or mini-sling placement [12]. Surgical management consisted of either local excision of the sling with vaginal closure or simple vaginal closure while preserving the sling in order to maintain its integrity and continence. Rare cases of sling erosion into the urethra or bladder have also been reported, often requiring surgical revision and more complicated management [14,15]. Additionally, labial or suprapubic granulomas secondary to foreign-body reaction around the sling material have been described [16], as well as chronic groin pain related to anchor tension or malposition, typically developing over weeks to months.

Both slings (Altis® and Abbrevo®) appeared to have similar subjective and objective cure rates and resolution of symptoms in the treatment of female SUI [9,17]. The long-term post-operative complications rate (i.e., de novo OAB (overactive bladder) symptoms and sling exposure) is similar in both procedures. Long-term outcomes of the Altis® single-incision sling after 10 years' follow-up showed only 5 out of 155 cases of mesh erosion and labial foreign-body reaction or nodule formation [18]. Similar results are reported in the recently published study by Mai Tu et al., which makes no reference to complications related to the anchoring arm of the sling [19]. This is the first case that describes nodule formation and inflammation of the anchoring arm of the Altis® sling with the simultaneous presence of groin pain, severe dyspareunia, and subepithelial vaginal erosion. Nodule formation and inflammation at the anchoring arm of the Altis® sling result from a chronic foreign body reaction. This reaction is likely triggered by persistent micro-motion of the rigid plastic anchor tip within the dynamic obturator tissues, preventing proper tissue integration. The ensuing mechanical irritation sustains a localized inflammatory cascade, leading to excessive fibroblast activity and collagen deposition. Ultimately, this process results in the encapsulation of the anchor within a palpable, painful fibrotic nodule in our patient. It is also critical to note that this complication, with its distinct mechanism, is not described in the current product information for the device.

The present case underscores the need for early recognition of atypical complications associated with single-incision polypropylene slings like Altis®. Clinically, early-onset groin or obturator pain after sling placement should prompt careful evaluation for anchor-related complications. Subepithelial erosion may be masked by granulation tissue, and visible mesh exposure may be absent. Imaging modalities, such as pelvic MRI or translabial ultrasound, have been reported as valuable tools to detect sling malposition, inflammation, or erosion, especially when physical examination is inconclusive [15,20]. Prompt identification of abnormal sling positioning or inflammatory changes allows timely surgical intervention, including excision of the affected sling arm and granulomatous tissue, resulting in symptom resolution and healing of the vaginal wall.

## Conclusions

This case highlights a previously unreported combination of very early-onset postoperative groin pain, subepithelial vaginal wall erosion masked by granulation tissue, paraurethral granuloma, and a labial subcutaneous inflammatory nodule following Altis® single-incision sling placement. Early recognition of atypical anchor-related complications is essential, even in the absence of visible mesh exposure. Prompt surgical management, including removal of the affected sling arm and excision of granulomatous tissue, can result in complete symptom resolution and restoration of vaginal wall integrity. Reporting such rare presentations contributes to a better understanding of mid-urethral sling complications and informs postoperative monitoring and patient counseling.
